# The Scientific Use of Alcohol

**Published:** 1907-08

**Authors:** 


### SELECTED ARTICLE.

### The Scientific Use of Alcohol.

*(From the Post-Graduate, May, 1907.)*

CONSIDERABLE excitement has occurred in England on account of the following pronounciamento

"In view of the statements frequently made as to present medical opinion regarding alcohol and alcoholic beverages, we, the undersigned, think it desirable to issue the following short statement on the subject—a statement which we believe represents the opinions of the leading clinical teachers as well as the great majority of medical practitioners.

"Recognising, that, in prescribing alcohol, the requirements of the individual must be the governing rule, we are convinced of the correctness of the opinion so long and generally held, that in disease alcohol is a rapid and trustworthy restorative. In many cases it may be truly described as life-preserving, owing to its power to sustain cardiac and nervous energy, while protecting the wasting nitrogenous tissues.

"As an article of diet we hold that the universal belief of civilised mankind that the moderate use of alcoholic beverages is, for adults, usually beneficial, is amply justified.

"We deplore the evils arising from the abuse of alcoholic beverages. But it is obvious that there is nothing, however beneficial, which does not by excess become injurious."

which appeared in the *Lancet* of March 30. It is signed by such men as McCall Anderson, William H. Bennett, James Crichton-Browne, W. R. Gowers, Jonathan Hutchinson and P. H. Pye-Smith, sixteen representative men in all. The correspondence that has been evoked in the *Lancet* is in the highest degree entertaining, although much of it exhibits very bad temper. But some of the most intemperate writers are the teetotalers.

The POST-GRADUATE rejoices in the bravery which has animated these distinguished British medical scientists. It is to be hoped that it will do a great deal of good in stemming the tide of prejudice against the moderate use of alcohol which constantly flows in the daily press and often in medical Journals. It is not too strong language to say that in disease, alcohol is a rapid and trustworthy restorative. It is certainly, also as said by these distinguished men, life preserving, owing to its power to sustain the energy of the heart and nervous system while protecting the wasting nitrogenous tissues.

In many parts of this country, there is no middle line between the saloon with its bar and its whiskey, and teetotalism. There are villages in the middle West particularly, where a decent citizen must perforce ally himself with the total abstainers, for it is either that or the use of whiskey as a beverage. We understand perfectly how such a condition of things makes teetotalers and crazy women run about and destroy saloons, but the belief of civilised mankind, as is stated in this document which we are reviewing, is that the moderate use of alcoholic beverages may be proper. It is proper food when taken with the meals, not for the young, but as was said years ago by George H. Lewes, "Alcohol is the milk of middle life and old age." It is a pity that the Anglo-Saxon and the Celt can only find proper stimulation in *strong* drink, which in the language of Scripture should be given to him who is ready to perish, but not as something to gladden the heart, for which wine is commended. It is a misfortune that the habit of drinking light wine and light beer with meals, cannot take the place of bar room whiskey drinking in this great country of ours. The medical profession has perhaps something for which to answer in not having educated the community at large, a little differently from what has been their habit. With the fact staring them in the face that most of the cultivated people of this country use alcohol as a beverage, we ought to be able, through the medical profession, to indicate to them the line the crossing of which becomes abuse. The late Chancellor Howard Crosby, received the severest condemnation on many sides, because he bravely taught the true doctrine of temperance and not total abstinence. To those who attach importance to the New Testament Scriptures, Dr. Crosby became unanswerable when he used to say that our Lord turned water into wine, and good wine, and at a wedding festival.

PEACE WITH A SWORD.

It is marvelous that we can have great Peace Congresses and the great men of the Old World and the New can excite themselves in haranguing about matters over which they have no control, but which belong to the Governments that represent the various peoples, while typhoid fever can almost decimate a neighboring city and threaten our own without any excitement being caused. Certainly there is an improper estimate of the relative importance of things, when the asphalt of the streets is left so as to endanger the horses feet and the people who they transport, and the streets are so dirty as to be a source of everlasting income to Oculists and Laryngologists, with typhoid fever raging all about our suburban borders and yet no general excitement is caused, while people are gathering by thousands to theorise and quarrel as to the day when men shall learn war no more. The POST-GRADUATE is heartily in favor of police and an army to bring Peace on the Earth. The greatest Authority for Peace, Himself stated that he did not come to bring Peace, but a sword. We shall need a sword for many a year to aid Governments to maintain cleanliness, order, justice and righteousness.

---

TREATMENT OF TETANUS BY SPINAL ANESTHESIA.—Dr. A. E. Russell reports in the *Lancet,* Sept. 23, 1905, on the successful treatment of tetanus by spinal anesthesia. Sixteen cc. of cerebral fluid were withdrawn and 3 cc. of the following solution injected: 1½ grains of beta-eucaine, 1-3 grain of morphin sulphate and 3 grains of sodium chlorid, with sufficient water to make 3½ ozs. This procedure was repeated four times. *Journal of the Amer. Med. Asso.*

